# Mechanical Properties of Composite Made from Bottom Ash Fractions of Municipal Waste Incineration Plant Products

**DOI:** 10.3390/ma18235302

**Published:** 2025-11-24

**Authors:** Maciej Tram, Katarzyna Sułkowska, Arkadiusz Jarosz, Andrzej Nowakowski

**Affiliations:** Strata Mechanics Research Institute of the Polish Academy of Sciences, Reymonta 27, PL–30-059 Krakow, Poland; maciej.tram@imgpan.pl (M.T.); katarzyna.sulkowska@imgpan.pl (K.S.); arkadiusz.jarosz@imgpan.pl (A.J.)

**Keywords:** bottom ash, thermal waste treatment, heat treatment, compressive strength, water permeability, circular economy, sustainable construction materials

## Abstract

**Highlights:**

**What are the main findings?**

- Bottom ash (BA) demonstrates strong potential for use as a secondary binder in sustainable construction materials.
- Smaller particle size increases compressive strength.
- Thermal treatment at 1000 °C improves binding activity and raises water permeability values.
- The mechanical performance of BA-based composites is primarily controlled by chemical activation.

**What are the implications of the main finding?**

- Bottom ash can be reused in sustainable construction.
- Proper pretreatment enhances the reactivity of BA-based composites.
- The results demonstrate that proper thermal treatment can transform BA into a reactive component suitable for cementitious systems.

**Abstract:**

The growing demand for sustainable and circular construction materials has increased interest in reusing by-products from municipal solid waste incineration. Due to the variability in the chemical composition of bottom ash (BA) between different plants, sampling seasons, and particle sizes, an individualized approach depending on its origin is essential. This study examines the potential of BA as a binding material in composite structures. Composites were prepared from BA fractions of different granulometries, including ground and thermally treated material. Compressive strength tests and structural analyses of density, porosity, and water permeability were performed to evaluate the influence of particle size and heat treatment on binding activity. The results show that smaller particle sizes significantly improved compressive strength, while the highest strength was obtained for samples calcined at 1000 °C, with an average increase of 84% compared to untreated material. Thermal treatment enhanced binding activity through the mobilization of hydration-active compounds bound in non-reactive mineral phases formed during water cooling and also increased water permeability due to the breakdown of porous structures. These findings confirm the potential of BA as a secondary binder in construction materials; however, further research is needed to improve its reactivity and mechanical performance.

## 1. Introduction

With the increasing amount of municipal solid waste generated, the problem of its storage and management has become more pronounced. One of the solutions to this issue is the thermal treatment of waste in municipal waste incineration plants. This process not only allows for a significant reduction in the volume of generated waste, but also enables the recovery of energy contained within it. Moreover, it fulfills the recommendations of the European Union arising from Council Directive 1999/31/EC on the landfill of waste [1] and Directive 2008/98/EC of the European Parliament on waste [2]. One of the facilities implementing thermal waste treatment processes is the municipal waste incineration plant in Kraków, operated by Krakowski Holding Komunalny S.A. (Krakow, Poland). It is among the most advanced installations of this type in Poland. The facility processes approximately 245,000 tons of municipal waste annually, generating about 100,000 MWh of electricity and 1,000,000 GJ of heat energy, which are supplied to the city’s energy network [3].

In the face of climate change and political pressure, it has become essential to seek new, more environmentally friendly energy sources, while increasing emphasis is placed on circular resource management. A portion of the energy generated during the combustion of waste containing biodegradable fractions can be classified as renewable energy [4,5]. Modern incineration plants employ the best available techniques, thus minimizing pollutants released into the environment as a result of municipal waste combustion. However, the combustion process produces residues that may account for up to approximately 30% of the feedstock mass [6]. These include fly ash (FA), bottom ash (BA), and solid residues from flue gas treatment [4,7]. Despite the reduction in the original waste volume, combustion residues still pose a significant challenge due to the necessity of their appropriate management and processing. Some of these residues, such as FA, are classified as hazardous waste, requiring specialized disposal methods [8]. They are categorized under waste code 19 01 13—fly ash containing hazardous substances. Nevertheless, due to their substantial volume, the more dominant fraction consists of bottom ash and furnace slags [9], which are classified as non-hazardous waste under code 19 01 12—bottom ash and slag other than those mentioned in 19 01 11 [10].

BA and furnace slag constitute approximately 80% of all by-products generated during the thermal treatment of municipal solid waste. Their safe management and reuse represent an important social and environmental challenge that requires urgent attention. BA is characterized by larger particle sizes, often reaching several tens of millimeters, and lower contents of chlorides and hazardous substances compared FA. The reuse of this material has been investigated for many years, and global interest in it continues to grow [11]. An increasing number of studies highlight the potential of bottom ash as a secondary raw material in construction, particularly in the production of composite and cement-based materials. This is primarily due to its mineral composition and physicochemical properties, which allow for the partial replacement of traditional binders [12,13]. Research has shown that appropriate thermal and mechanical treatment of BA can improve its performance characteristics, making it an attractive alternative to conventional cementitious materials while aligning with the principles of the circular economy [14]. Current efforts include, among others, the production of hybrid cements through the alkaline activation of clinker–ash mixtures [13], the use of BA and FA as additives in concrete and road sub-base layers [7,15], and the development of innovative eco-cement concepts activated by carbonation, which can achieve properties comparable to those of Portland cement while exhibiting a significantly lower carbon footprint [4,16]. Bottom ash also shows potential for use as a filling material in salt mine voids, where its physical and binding properties could support both structural stability and environmental protection [17]. Moreover, novel applications are attracting growing attention, such as the use of ashes in 3D printing technology [18], and in the synthesis of geopolymer materials [14].

An increasing number of laboratory studies focus on analyzing the mechanical properties of composite materials containing BA, both as aggregate and as a binding component. Mechanical strength tests typically determine compressive, tensile, and flexural strength as well as the modulus of elasticity, which allows assessment of BA’s potential as a substitute for conventional mineral raw materials [19,20]. Sirico et al. (2024) [21] demonstrated that the use of ground BA in cement mortars enables maintaining or even improving strength compared to reference samples, provided that the raw material is pretreated to reduce the content of reactive metals. In their study, the authors showed that incorporating BA as a mineral admixture can enhance the ductility and corrosion resistance of mortars, despite a slight decrease in compressive strength. In the context of road applications, untreated BA reaches California Bearing Ratio (CBR) values of 50–80% under dry conditions and 37–63% after soaking [22]. It has also been shown that aging and carbonation processes in BA contribute to improved volumetric stability and mechanical properties of mixtures, while reducing the risk of expansion caused by the presence of metallic aluminum [23].

The properties and, consequently, the utilization potential of BA are strongly dependent on its chemical composition. Qualitatively, similarly to its mineral composition, the chemical composition of BA is generally comparable regardless of the country or the specific incineration plant. However, significant quantitative variations are observed between plants, particle-size fractions, and even sampling seasons. The main component of BA is amorphous silica, originating from glass present in the processed waste, with its mass fraction often exceeding 50% [24]. The second most common component is calcium oxide (CaO), whose content may also exceed 50% [25], although this is typically associated with lower SiO_2_ and Al_2_O_3_ contents. A comparison of BA chemical compositions from different European countries shows noticeable variations: 37.2% SiO_2_, 24.6% CaO, and 9.30% Al_2_O_3_ for Spanish BA [26]; approximately 47% SiO_2_, 18% CaO, and 9.50% Al_2_O_3_ for Italian material [27]; and 52.9% SiO_2_, 13.44% CaO, and 10.18% Al_2_O_3_ for BA from the Netherlands [28]. The same Spanish authors, both in that publication and in subsequent works, reported the following BA compositions: 45.44%, 52.08%, and even 64.5% SiO_2_; 17.55%, 20.72%, and 14.7% CaO; and 10.38%, 6.35%, and 4.97% Al_2_O_3_ [26,29,30]. Similarly, published data on BA from China show a comparable range of variability, with SiO_2_, CaO, and Al_2_O_3_ contents ranging from 21.68 to 53.82%, from 14.44 to 54.21%, and from 5.76 to 14.18%, respectively [25,31,32,33]. The quantitative composition of BA also varies depending on the sampling period. Studies of BA collected from the same waste-to-energy plant in Japan at different times of the year revealed significant variations of 25.88–40.26% SiO_2_, 23.23–37.55% CaO, and 15.29–17.79% Al_2_O_3_ [34]. Even material sampled simultaneously from a single facility can differ in chemical composition depending on particle size. As particle size decreases, the proportion of CaO increases at the expense of SiO_2_, from 40.9% and 18.3% for SiO_2_ and CaO in the 2.5–4.0 mm fraction to 14.8% and 47.6% for SiO_2_ and CaO in the <0.025 mm fraction [35].

Such a wide range of variability highlights the lack of a clear classification and justifies distinguishing between calcium-rich and silica-rich ashes. The variability of chemical composition has a crucial impact on the binding and mechanical properties of BA. The proportions of the four main oxides determine the course of hydration processes and the formation of gels responsible for strength development in alkali-activated materials [20,36,37]. As emphasized by Chen et al. (2024) [20] the presence of an amorphous phase rich in Si, Ca, and Al, which accounts for more than 50% of BA mass, plays a key role in its reactivity, whereas an excessive amount of metals such as metallic aluminum can cause undesirable foaming and a reduction in material strength. Therefore, an individualized approach to the material depending on its origin becomes essential.

In this study, the authors present an investigation of BA obtained from The Thermal Waste Treatment Plant in Krakow in the context of its use as a binding material and the analysis of the effects of its repeated thermal treatment. The selected series, differing in particle size or treatment method, were subjected to compressive strength testing and comparative analysis of the water filtration coefficient. The influence of the selected fraction and applied modification on the strength parameters and porosity of the prepared composites was also evaluated. The originality of the study stems from the simultaneous analysis of the influence of two independent factors on the reactivity and binding properties of composites produced with BA. These factors are

- The grain-size fraction used in the composite, which reflects the structural characteristics of the material;
- Exposure of the material to high temperatures, which represents an attempt to identify a method for improving its properties in the context of potential applications.

This approach was motivated by the need to explore new ways of utilizing BA in accordance with the principles of the circular economy.

## 2. Materials and Methods

### 2.1. Material Origin and Preliminary Identification

**Figure 1:**
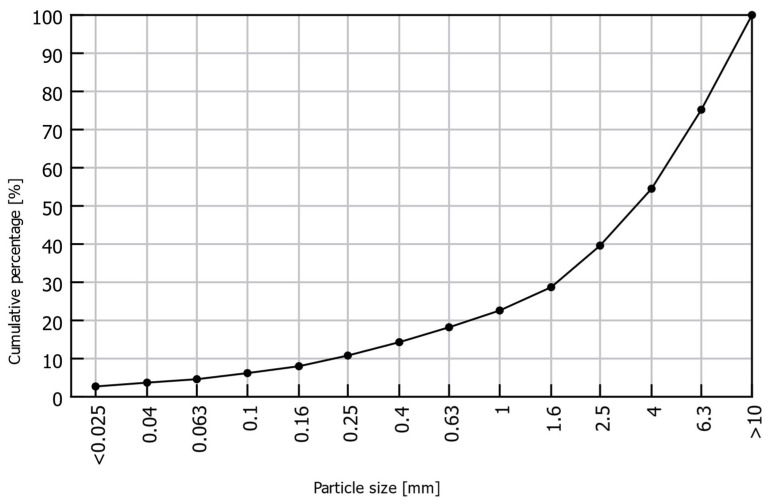
Particle size distribution of BA.

The material used in this study was obtained from the Thermal Waste Treatment, Kraków, Poland. It was produced through the oxidation of waste at a temperature of 1000 °C and subsequently cooled with water [38]. The material was delivered in several containers in a mixed form. The granulometric analysis presented in Figure 1 shows that a significant portion of the BA consisted of coarse gravel fractions, among which the fraction with the largest screened particle size (>10 mm) had the highest mass share. The total gravel fractions (>2.5 mm) accounted for as much as 60% of the analyzed BA. According to the available literature, the optimal particle size distribution for Portland cement consists of 90% material with a fraction <63 µm [39]; however, this fraction represented only 3.7% of the total BA, which greatly limits its usable amount. Therefore, to utilize a larger portion of the processed material, the strength tests included not only the <63 µm fraction, designated as sample series BA63-UG, but also the ground bulk material of the <63 µm fraction (sample series BA63-G) and two coarser BA fractions, <200 µm and <400 µm (designated as sample series BA200-UG and BA400-UG, respectively). The selected coarser fractions accounted for approximately 8.0% and 10.8% of the total material, respectively.

**Figure 2:**
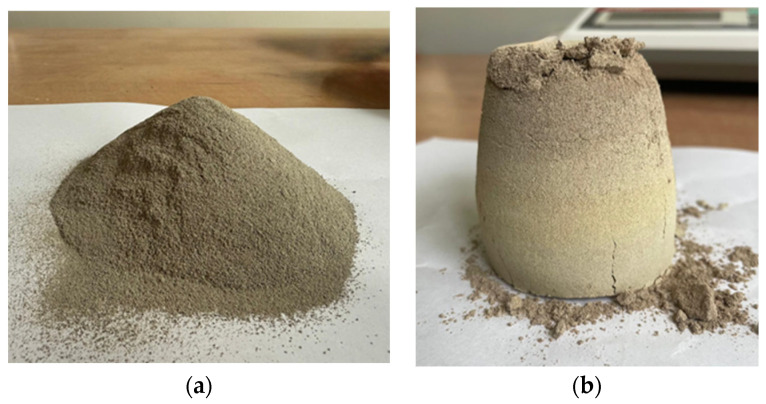
Bottom ash with particle size below 63 µm before calcination (**a**) and after calcination at 1000 °C (**b**).

The cooling process applied at the incineration plant to rapidly reduce the temperature of the processed waste has a significant impact on the properties of BA [40]. It leads to changes in the morphology of the base material as well as its chemical composition, resulting in the formation of calcium- and aluminum-bearing compounds (i.e., ettringite, portlandite, and Friedel’s salt) [41]. Additionally, the presence of calcium carbonate (CaCO_3_) in the material suggests [42] that exposure of the hot and moistened waste to air containing carbon dioxide may have induced carbonation reactions. To eliminate the aforementioned compounds, the authors of this study decided to reheat the investigated material at 1000 °C. According to the literature, this temperature is sufficient to decompose these mineral phases. Friedel’s salt decomposes within the range of 310–385 °C [43], whereas portlandite decomposes at approximately 450–550 °C [44]. The calcination process was carried out using a tiled furnace. The thermal modification was performed on unground material with a particle size <63 µm, which was heated at the target temperature for 1 h and then cooled together with the furnace. The material processed in this way was designated as sample series BA63-HT. A visual comparison of BA before and after the thermal treatment is presented in Figure 2. As a result of this process, the mass loss of the calcined material reached 27.7%, while its specific density increased from 2.46 for the untreated material to 3.03 after calcination.

Table 1 summarizes all selected BA series together with information on the applied particle-size fraction and processing method. Series using unmodified material were marked with the postfix UG (unground), while additionally processed BA was assigned the postfix G (grounded) or HT (thermally treated). The BA-G series was obtained by additionally grinding the entire BA material using a 3000 g laboratory mill equipped with a clamping closure system. The grinding process lasted approximately 5 min at a speed of 36,000 rpm, which enabled the production of a finer grain size and allowed an adequate amount of material <63 µm to be sieved out. During operation, the device was gently tilted by adjusting the angle of the grinding chamber, which promoted uniform fragmentation and prevented material from accumulating on the chamber walls. Furthermore, Table 2 presents the chemical compositions of the respective series. The quantitative content of chemical compounds in BA varied depending on the particle size and modification method. With decreasing particle size, the percentage of SiO_2_ continuously decreased. The finer material contained higher CaO content, showing greater similarity to the chemical composition of Portland cement [45].

### 2.2. Compressive Strength Tests and Structural Properties

The dimensions and shape of standard concrete specimens used in strength testing are defined in the Polish Standard PN-B-06265 [47]. According to this standard, such specimens should be cubic with a side length of 100.0 mm, assuming that the maximum grain size in the concrete d*_max_* does not exceed 16.0 mm. However, a review of the literature on composites produced using BA showed that much smaller specimens were often used for testing; for example, [26] employed cubes with a 25.0 mm edge, while [48,49] used cubes with a 50.0 mm edge. Therefore, the authors considered it acceptable to perform tests on cubic samples with an edge length of 56.0 mm.

The composite was prepared from a mixture of BA with a specified granulation and technical sand (TS), combined in defined weight ratios and mixed with water to achieve a consistency that allowed convenient filling of molds without the risk of leakage. The following BA/TS ratios were used: 1:2, 1:1, 2:1, and 3:0. Table 3 presents an overview of all tested samples, specifying the percentage share of BA in the composite and the water content. The amount of water required to obtain the desired consistency increased as the BA particle size decreased. Additionally, calcination also resulted in higher water absorption of the material.

**Figure 3:**
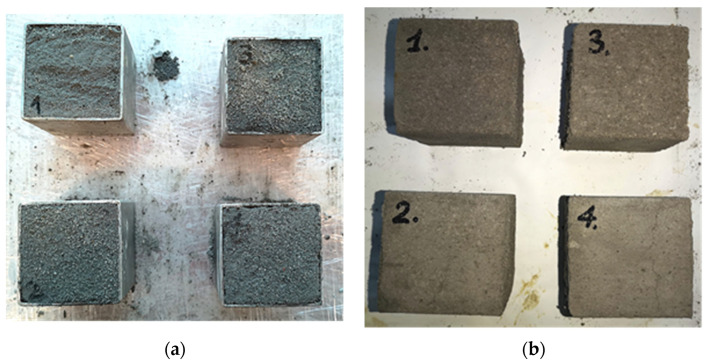
Preparation of samples for testing: samples at the moment of composite casting into molds (**a**), and samples removed from molds after 21 days (**b**); the numbers visible on the samples are the sample numbers within the same series.

The molds filled with the material were placed for 21 days in a sealed container with a water-filled bottom to maintain constant humidity during the composite curing process. After 21 days, the samples were removed from the molds and left in the laboratory at room temperature for 7 days. Figure 3 shows the prepared samples in the molds at the time of casting and the same samples after 21 days, once removed from the molds.

**Figure 4:**
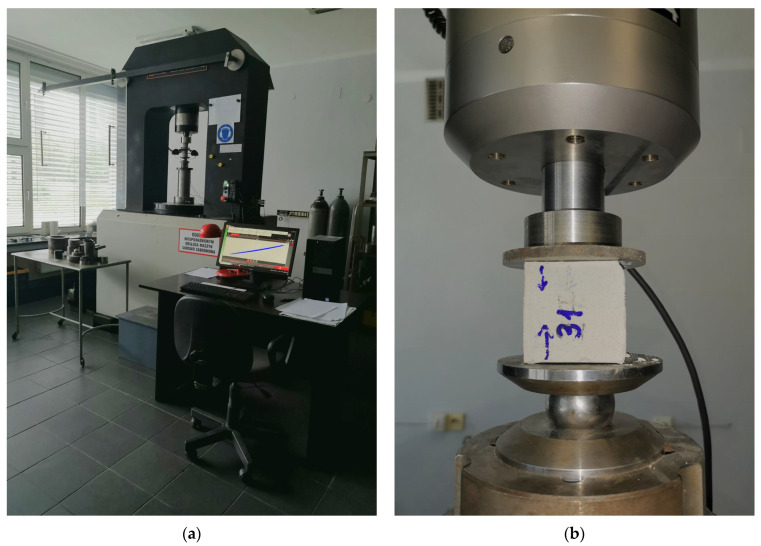
Composite sample during the uniaxial compression test: view of the testing setup (**a**) and sample during the experiment (**b**).

On the 28th day after casting, the compressive strength of the composite samples was tested using a uniaxial compression test. The tests were carried out with an INSTRON 8500/8800 Rock Testing System (Norwood, MA, USA). The testing setup and a sample during the experiment are shown in Figure 4. During the test, the sample was compressed at a constant rate of axial stress increase, which was set at 0.5 MPa/s.

Among the structural properties, the composite’s bulk density (*ρ*), specific density (*ρ*_s_), and porosity (*n*) were examined. Porosity was calculated based on the measured bulk and specific densities using the well-known formula

(1) $$n=1-\frac{\rho }{\rho_{s}},$$

The bulk density of each sample was determined by dividing its mass (*m*) by its volume (*V*), which was calculated from its dimensions. The specific density was measured using a Micromeritics AccuPyc II 1340 helium pycnometer (Norcross, GA, USA).

### 2.3. Water Permeability Coefficient

The permeability coefficient tests were carried out using measurement equipment manufactured by Fröwag (Obersulm, Germany). The setup consisted of a triaxial permeameter system with flexible walls, allowing the determination of soil permeability coefficients under confining pressures up to 2.5 bar, in accordance with the standard [50]. This apparatus enabled simultaneous testing of up to three samples, each placed in a separate pressure chamber. The pressure difference *ΔP* was adjusted using two manometers.

**Figure 5:**
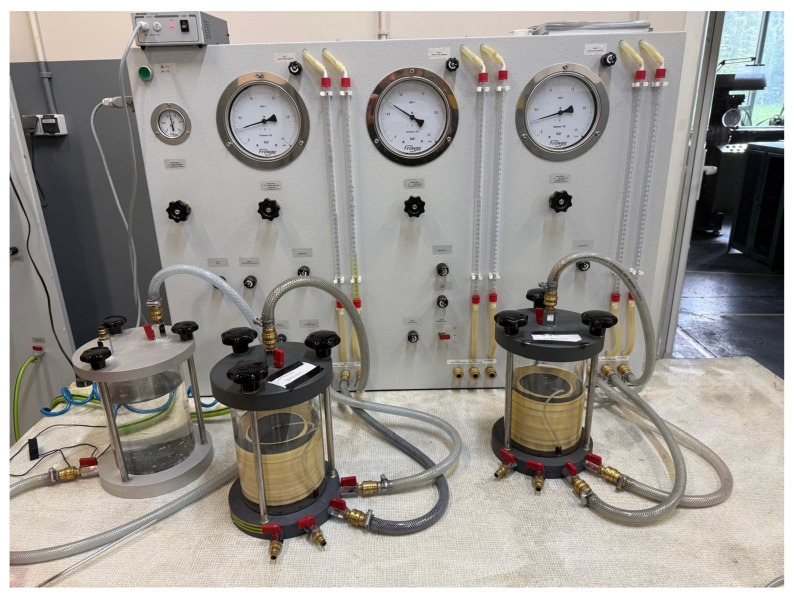
Test setup for measuring the water permeability coefficient with mounted samples.

The water permeability coefficient was determined for samples made of BA with a particle size <63 µm, both before and after thermal modification, i.e., BA63-UG and BA63-HT. Cylindrical samples with a diameter of 100 mm were mounted in the apparatus, as shown together with the test setup in Figure 5. The samples were prepared by mixing the raw material with water and compacting it under a load of 2 kN using the previously described INSTRON 8500/8800 testing machine. The height of both samples was 56 mm, and the applied constant pressure differences were 0.8 bar (8.158 mH_2_O) and 0.3 bar (3.059 mH_2_O) for BA63-UG and BA63-HT, respectively. Measurements were performed under three different confining pressures: 1, 1.5, and 2 bar. The total filtrated volume (*Q*) was determined based on readings from outlet burettes. Each measurement point represented 10 individual readings taken in the upward flow direction. The permeability coefficient *k*, expressing the relationship between the hydraulic gradient and the water flow velocity, was calculated using the following equation:

(2) $$k=\frac{Q\cdot l}{A\cdot \Delta h\cdot t}\left[\frac{m}{s}\right],$$

where

*l*—sample height [m];
*Q*—filtrated volume [m^3^];
*Δh*—hydraulic head difference [m];
*A*—cross-sectional area of the sample [m^2^];
*t*—measurement duration [s].

## 3. Results

### 3.1. Structural and Compressive Strength Properties

**Figure 6:**
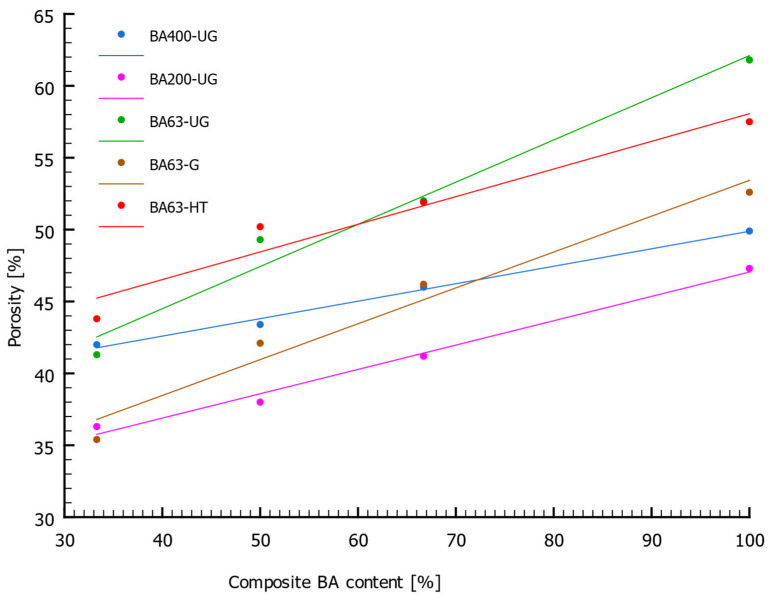
Relationship between the porosity (*n*) of the composite and the BA content.

The results of the compressive strength (*R_c_*), density, and porosity measurements of the composite samples made using specific BA fractions and BA calcined at 1000 °C are summarized in Table 4. The relationship between the porosity of the composite samples and BA content is graphically illustrated in Figure 6. The presented results suggest that the porosity of the samples does not depend on the method of BA selection or its particle-size fraction. However, the percentage share of BA in the composite composition is a significant factor, as porosity increases proportionally with higher BA content. Similarly, BA calcination at 1000 °C proved to have no significant influence on porosity, showing no clear trend of change. Composites containing coarser BA particles exhibited slightly lower porosity values. This can be explained by differences in particle morphology. Finer particles (<63 µm) contain a greater number of micropores, which increases their water absorption capacity and leads to higher total porosity. In contrast, coarser particles are characterized by a more glassy structure formed during the rapid cooling of BA.

**Figure 7:**
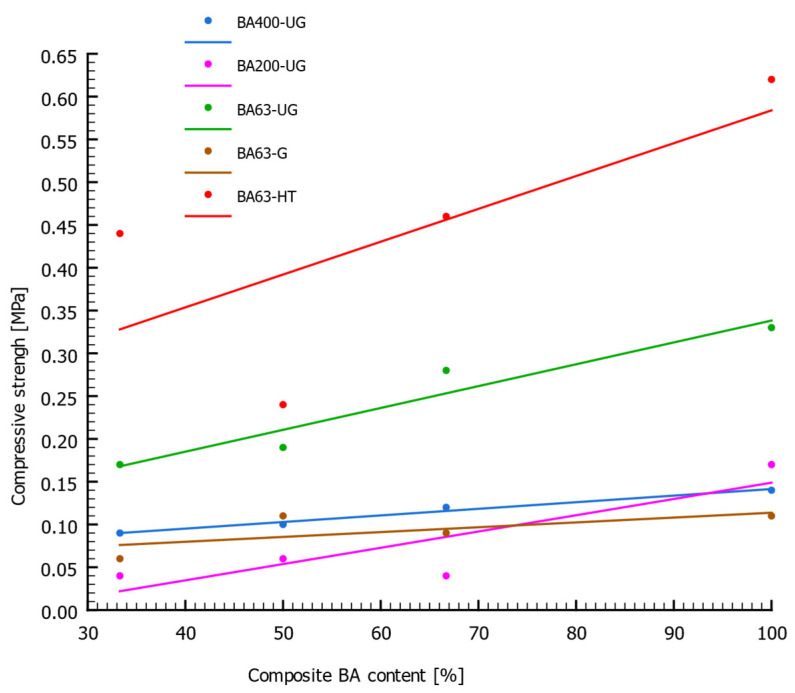
Relationship between the compressive strength (*R_c_*) of the composite samples and the BA content.

Figure 7 presents the relationship between the compressive strength of the samples and the percentage content of BA. Within each measurement series, the *R_c_* value increased with higher BA content, and the highest *R_c_* values were obtained for samples made entirely of BA (without TS admixture). In absolute terms, the highest compressive strength was recorded for the BA63-HT samples, which were prepared from calcined material. The results show a significant increase in compressive strength, which is inversely proportional to BA particle size. The *R_c_* parameter increased with decreasing particle size, showing a rise of 21.4% and 135.7% for BA200-UG/4 and BA63-UG/4, respectively, compared with the coarsest BA400-UG/4 composite. The BA63-G series exhibited very low binding capacity and a weaker correlation between *R_c_* and BA content, despite using material with the finest grain size. This effect most likely results from chemical composition changes in the ground material. The compressive strength analysis of individual series correlates with the previously presented relationship (see Table 2) between particle size and the content of compounds involved in the binding process. Although the BA400-UG and BA200-UG series have a chemical composition closer to that of Portland cement (compared with BA63-G), they consist of much larger grains, with the fine fraction (<63 µm) accounting for approximately 18.9% and 45.3% of their total mass, respectively. The BA63-G series contained a significantly higher proportion of silica compared to the other composites, making it chemically more distinct from Portland cement. This is understandable, as finer silica-rich particles were added to grains with a more favorable ratio of binding compounds. Although silica participates in hydration reactions, it is less desirable in this context. In the silicate phases of Portland cement clinker, CaO compounds are approximately 2.5 times more desirable than SiO_2_ compounds [39]. Additionally, during the curing of the BA63-G specimens, noticeable swelling was observed. This phenomenon may potentially result from reactions between amorphous SiO_2_ and alkalis such as potassium and sodium. Such reactions are known to occur in concrete mixtures containing aggregates with amorphous silica, leading to the formation of alkali–silica gel. This gel exhibits strong water absorption accompanied by an increase in volume, which induces internal stresses that may cause mechanical damage to the binding matrix [51]. The XRD analyses of ground BA presented by Malaiškienė et al. (2023) [52] confirmed the presence of amorphous SiO_2_ phases. A similar swelling effect was not observed for the other analyzed series, which may indicate that mechanical processing of BA facilitated the release of amorphous silica phases. As a result, the overall content of binding compounds in the composite mass was substantially reduced.

**Figure 8:**
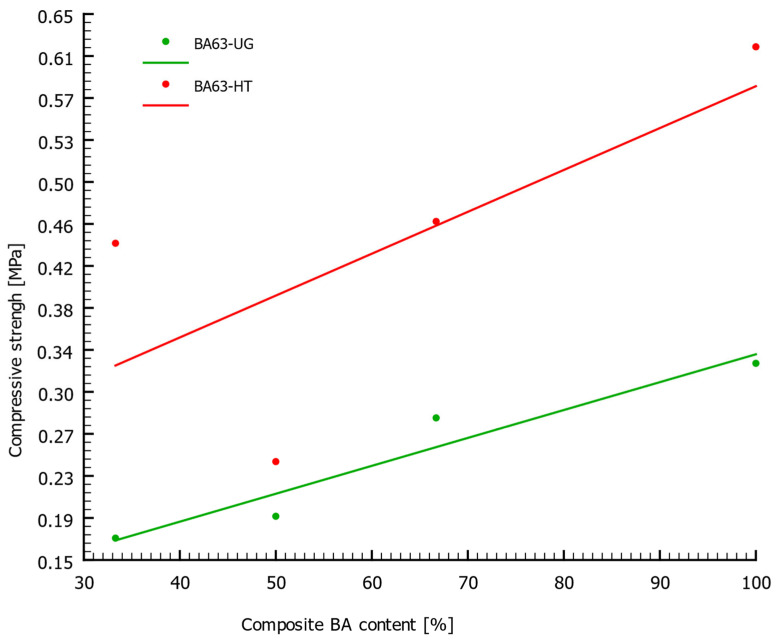
Relationship between the compressive strength (*R_c_*) of the composite samples and the BA content for the <63 µm fraction before and after thermal treatment.

Figure 8 presents the *R_c_* results for BA < 63 µm before and after thermal modification. The data indicate that calcining BA at 1000 °C significantly increased the compressive strength of the prepared composite samples. The greatest improvement was observed for the sample with the lowest BA content, where compressive strength increased by as much as 159%. The average *R_c_* improvement across the entire series was 84%. The shape of the compressive strength-versus-BA content curve did not change significantly, suggesting that the calcination process did not strongly affect this correlation. The observed increase in strength is likely related to the decomposition of compounds in which CaO is bound (e.g., CaCO_3_), thus making them unavailable for hydration reactions. The presented curves confirm the effectiveness and justification of the applied thermal modification.

### 3.2. Water Permeability Coefficient Testing

**Figure 9:**
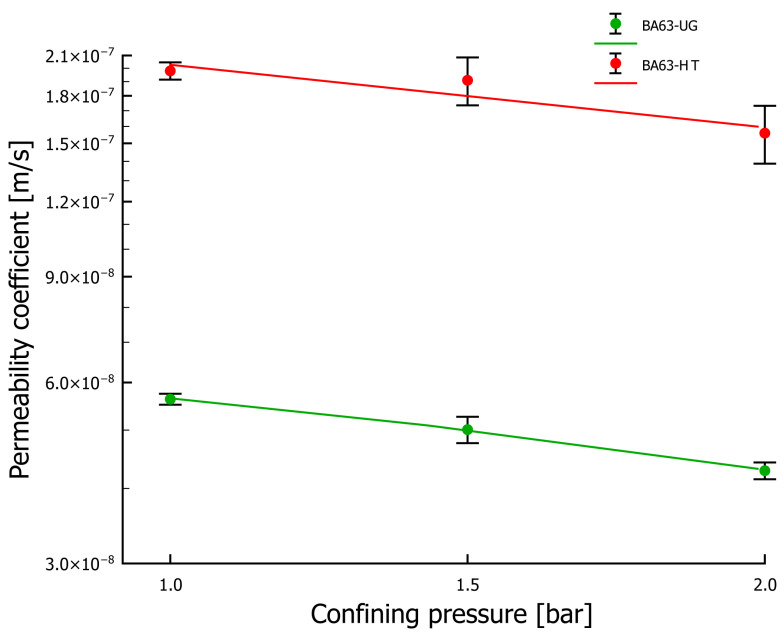
Water permeability coefficients for BA samples with a <63 µm fraction before and after thermal treatment.

A comparative analysis of the water permeability coefficient was conducted for the BA63-UG and BA63-HT samples. The measurement results with marked error bars are presented in Figure 9. Both samples exhibited a slight tendency toward a decrease in the water permeability coefficient with increasing confining pressure. This may indicate further compaction of the samples under the pressure exerted on the walls of the flexible sleeve in which they were placed during testing. The permeability coefficients obtained for BA63-UG were lower by an order of magnitude, corresponding to a 71.6–73.8% reduction compared with BA63-HT across the tested range of confining pressures. The small difference in relative change values, approximately 2.2 percentage points, suggests that both samples were initially compacted to a similar degree.

## 4. Discussion

The porosity of all prepared composites ranged from 35.4% to 61.8%, which is considerably higher than that of pure BA, whose total porosity is 24.3% [53]. In cement-based samples, compressive strength decreases significantly with increasing porosity [54], suggesting that reducing porosity could positively affect the quality of the prepared samples. The obtained compressive strength results confirm that both the mechanical and structural properties of BA-based composites depend on particle size and thermal treatment. This observation is consistent with the findings of Bouzar et al. (2024) [55], who demonstrated that eco-binders produced from secondary raw materials exhibit increased reactivity and improved mechanical properties when appropriate pre-treatment methods are applied. Reducing particle size led to an improvement in compressive strength, which may indicate greater reactivity of smaller grains. For the BA400-UG sample, *R_c_* increased from 0.09 MPa at 33.3% BA content to 0.14 MPa at 100% BA. For BA200-UG, these values were 0.04 MPa and 0.17 MPa, while for BA63-UG, *R_c_* increased from 0.17 MPa to 0.33 MPa. The highest compressive strength was achieved for the BA63-HT/4 series (0.62 MPa), corresponding to an increase of approximately 87.9% compared to the BA63-UG/4 sample (0.33 MPa). The average strength increase within the BA63-HT series relative to the uncalcined material was approximately 84%, confirming the significant influence of calcination on the binding properties of the material.

A similar increase in compressive strength resulting from thermal modification of BA was reported for the specimens examined by Chai et al. (2025) [56]. After 28 days of curing, the samples calcined at 600 °C showed an improvement of approximately 55.1%. Alkali-activated materials also exhibit an increase in *R_c_* after thermal treatment of the precursor. The effect of BA modification at a comparable temperature was presented by Huang et al. (2020) [32]. They showed that preliminary thermal treatment at 1050 °C improved compressive strength from 2.4 MPa for the untreated sample to 10.4 MPa for the thermally modified one. In their study, the authors demonstrated that the best strength results for mortars without additives were obtained for composites cured at 700 °C and subjected to prior alkali defoaming.

The presented trend of increasing compressive strength is consistent with other studies showing that reducing BA particle size enhances its reactivity and ability to form hydration phases. Malaiškienė et al. [52] demonstrated that grinding BA to a particle size below 75 µm results in cement mortars with compressive strength comparable to or higher than that of reference samples, while also reducing the content of reactive metals. However, in this study, mechanical grinding of BA led to a decrease in compressive strength; in the BA63-G/4 sample, *R_c_* was only 0.11 MPa, representing a 67% reduction compared to the BA63-UG series.

Despite the significant relative increases in *R_c_*, the absolute strength values of the obtained composites remain well below the thresholds even for the lowest cement classes defined by the standard [57]. This indicates the need for further research and consideration of suitable activators that could substantially improve mechanical performance. Data from the literature confirm that the use of appropriate alkaline solutions can produce a cement-like composite with strength properties comparable to those of pure Portland cement [26,48,58]. Likewise, increasing the calcination temperature to the clinker firing temperature of Portland cement may positively influence the analyzed material. In this process, at temperatures above 1100 °C, secondary silicate phases (2CaO·SiO_2_) are formed, which further react at 1300 °C to produce tricalcium silicate (3CaO·SiO_2_) and tetracalcium aluminoferrite (4CaO·Al_2_O_3_·Fe_2_O_3_) [45].

Permeability tests of the prepared samples showed the highest coefficient for the BA63-HT sample tested under a confining pressure of 1 bar, reaching $1.98\times 10^{-7}m/s$. The lowest permeability was recorded for the BA63-UG sample at a confining pressure of 2 bar, with a value of $4.28\times 10^{-8}\;m/s$. W The higher water permeability coefficients obtained for the calcined material may be linked to changes in mineral composition. It is likely that calcination caused the breakdown of highly porous structures (e.g., CaCO_3_), leading to the observed improvement, although this hypothesis requires further verification.

The obtained results were compared with data from the literature for various materials compiled by [59]. In the context of non-cohesive materials, the measured values fall within the range typical of glacial clays or silty sands. When compared to rocks, the tested material can be classified as sandstone, limestone, or dolomite. Compared with other wastes, such as coal ash from thermal power plants, BA63-HT exhibited similar water permeability for samples of comparable compaction, approximately $4.00\times 10^{-7}m/s$ for coal ash and $1.98\times 10^{-7}m/s$ for BA63-HT [60]. In contrast, when compared with unprocessed municipal waste (before combustion at the waste-to-energy plant), the obtained water permeability coefficient was significantly lower by up to two orders of magnitude under the same confining pressures [61]. A comparison with available BA studies analyzing water permeability for mixed materials shows that the presented experimental results are up to four orders of magnitude lower [62]. The results reported by Ogunro et al. (2004) [63] for mixed BA material compacted to about 95% exhibited permeability values in the range of $\left(68.3-4.63\right)\times 10^{-5}\;m/s$. The significantly lower permeability observed in the presented samples with finer particle size may be attributed to improved packing density, which enhances the filling of available pore spaces within the sample cross-section.

## 5. Conclusions

The conducted research demonstrated that BA originating from The Thermal Waste Treatment Plant in Krakow has potential to be valorized as a secondary raw material for use as a binder in construction materials, provided that the raw material is properly prepared. It was found that particle size has a significant influence on the mechanical properties of the analyzed composites since compressive strength (*R_c_*) clearly increases as particle size decreases. For fractions smaller than 63 µm, considerably higher *R_c_* values were observed compared with composites composed of larger fractions, which confirms that grain size reduction supports binding processes and improves material cohesion. The best results were achieved for samples prepared from material calcined at 1000 °C. These composites exhibited markedly higher compressive strength than the other series, which indicates that thermal treatment significantly enhances the binding activity of BA. This effect primarily results from the elimination of unstable mineral phases and the increase in reactive calcium oxide, which promotes the formation of stable binding phases that strengthen the composite structure.

Mechanical grinding of the material without additional chemical activation led to a decrease in strength, which is associated with a change in the proportions of major oxides with an increase in SiO_2_ content and a decrease in CaO content. Although porosity increased with higher BA content, the best results were obtained for composites made from calcined material, which demonstrates that chemical activity rather than pore structure plays the dominant role in determining strength. The observed increase in compressive strength is likely the result of two contributing effects: the restructuring of the pore network and the enhanced reactivity of BA after calcination. Despite these benefits, the relatively low strength and incomplete reactivity of BA still represent major limitations to its potential use in construction materials.

The authors emphasize, however, that this research is still ongoing and that the obtained results will be further developed. Future stages of this study will include experiments aimed at improving the reactivity and mechanical performance of the material through, among other processes, alkaline activation and calcination at higher temperatures, up to 1450 °C, which will enable better control of crystallization processes and the removal of phases inactive in binding. Further investigations will help determine how BA can be effectively utilized as a low-energy, sustainable secondary raw material in modern construction materials and how its processing parameters can be optimized to maximize performance while minimizing environmental impact.

The aim of the research presented in this work was to identify possibilities for the reuse of residues produced by municipal waste incineration plants. The advantage of this approach is the potential to convert waste accumulated at plant storage sites into a useful and reusable material. The obtained results indicate possible limitations arising, for example, from the incomplete reactivity of BA, which prevents achieving sufficiently high compressive strength in the produced composite.

This study presents an alternative pathway for the valorization of BA as a potential binding material. The experiments demonstrated that the reactivity of BA can be enhanced not only by chemical methods but also through appropriate physical and thermal treatment. Attention was also drawn to the relationship between the reactivity of BA used in the composite and its particle size distribution. The presented results should support the development of new methods for utilizing the products of municipal waste incineration.

## Figures and Tables

**Table 1:** Selected BA series with information on the applied particle-size fraction and processing method.

Sample Series	Fraction Size	Treatment
BA400-UG	<400 µm	unground
BA200-UG	<200 µm	unground
BA63-UG	<63 µm	unground
BA63-G	<63 µm	ground
BA63-HT	<63 µm	unground, thermally treated at 1000 °C

**Table 2:** Chemical composition of selected sample series and an example composition of Portland cement [46].

Specimen Name	SiO_2_	CaO	Al_2_O_3_	Fe_2_O_3_	SO_3_	MgO	K_2_O	Cl
	wt%							
Portland cement	21.62	62.12	2.37	1.51	2.03	1.57	0.84	0.097
BA400-UG	17.30	38.45	4.75	2.47	10.56	1.88	1.08	2.24
BA200-UG	15.67	41.52	4.51	2.40	10.81	1.90	1.07	2.27
BA63-UG	19.72	46.01	11.57	3.72	9.02	3.05	1.14	2.75
BA63-G	29.93	28.13	5.60	2.70	7.36	1.83	1.14	1.51
BA63-HT	22.43	49.16	8.29	3.29	8.73	2.01	0.90	1.90

**Table 3:** Tested samples with corresponding BA content and water percentage.

Sample Series	Sample Number	BA Content [%]	Water Content [%]
BA400-UG	1	33.3	21.6
	2	50.0	23.4
	3	66.7	29.4
	4	100	37.0
BA200-UG	1	33.3	16.8
	2	50.0	14.5
	3	66.7	18.3
	4	100	24.2
BA63-UG	1	33.3	31.4
	2	50.0	42.6
	3	66.7	52.4
	4	100	80.8
BA63-G	1	33.3	15.4
	2	50.0	17.9
	3	66.7	21.9
	4	100	33.9
BA63-HT	1	33.3	32.0
	2	50.0	45.3
	3	66.7	50.4
	4	100	92.0

**Table 4:** Results of compressive strength (*R_c_*) bulk density (ρ), specific density (ρ_s_), and porosity (*n*) of the prepared composites.

Serial Name	Sample Number	*R_c_* [MPa]	*ρ* [g/cm^3^]	*ρ_s_* [g/cm^3^]	*n* [%]
BA400-UG	1	0.09	1.52	2.61	42.0
	2	0.10	1.46	2.58	43.4
	3	0.12	1.38	2.55	46.0
	4	0.14	1.24	2.48	49.9
BA200-UG	1	0.04	1.67	2.63	36.3
	2	0.06	1.62	2.62	38.0
	3	0.04	1.53	2.60	41.2
	4	0.17	1.36	2.58	47.3
BA63-UG	1	0.17	1.52	2.58	41.3
	2	0.19	1.29	2.55	49.3
	3	0.28	1.21	2.52	52.0
	4	0.33	0.95	2.48	61.8
BA63-G	1	0.06	1.70	2.64	35.4
	2	0.11	1.52	2.63	42.1
	3	0.09	1.41	2.62	46.2
	4	0.11	1.23	2.59	52.6
BA63-HT	1	0.44	1.51	2.69	43.8
	2	0.24	1.35	2.71	50.2
	3	0.46	1.31	2.72	51.9
	4	0.62	1.17	2.76	57.5

## Data Availability

The original contributions presented in this study are included in the article. Further inquiries can be directed to the corresponding author.
